# Imaging and the completion of the omics paradigm in breast cancer

**DOI:** 10.1007/s00117-018-0409-1

**Published:** 2018-06-08

**Authors:** D. Leithner, J. V. Horvat, R. E. Ochoa-Albiztegui, S. Thakur, G. Wengert, E. A. Morris, T. H. Helbich, K. Pinker

**Affiliations:** 1 Department of Radiology, Breast Imaging Service, Memorial Sloan Kettering Cancer Center, 300 E 66th St, 7th Floor, 10065 New York, NY USA; 2 Department of Diagnostic and Interventional Radiology, University Hospital Frankfurt, Frankfurt, Germany; 3 Department of Biomedical Imaging and Image-guided Therapy, Division of Molecular and Gender Imaging, Medical University Vienna, Vienna, Austria

**Keywords:** Breast neoplasms, Magnetic resonance imaging, Diffusion-weighted imaging, Biomarkers, Gene expression, Molecular subtypes, Brustneoplasien, Magnetresonanztomographie, Diffusionsgewichtete Bildgebung, Biomarker, Genexpression, Molekulare Subtypen

## Abstract

Within the field of oncology, “omics” strategies—genomics, transcriptomics, proteomics, metabolomics—have many potential applications and may significantly improve our understanding of the underlying processes of cancer development and progression. Omics strategies aim to develop meaningful imaging biomarkers for breast cancer (BC) by rapid assessment of large datasets with different biological information. In BC the paradigm of omics technologies has always favored the integration of multiple layers of omics data to achieve a complete portrait of BC. Advances in medical imaging technologies, image analysis, and the development of high-throughput methods that can extract and correlate multiple imaging parameters with “omics” data have ushered in a new direction in medical research. Radiogenomics is a novel omics strategy that aims to correlate imaging characteristics (i. e., the imaging phenotype) with underlying gene expression patterns, gene mutations, and other genome-related characteristics. Radiogenomics not only represents the evolution in the radiology–pathology correlation from the anatomical–histological level to the molecular level, but it is also a pivotal step in the omics paradigm in BC in order to fully characterize BC. Armed with modern analytical software tools, radiogenomics leads to new discoveries of quantitative and qualitative imaging biomarkers that offer hitherto unprecedented insights into the complex tumor biology and facilitate a deeper understanding of cancer development and progression. The field of radiogenomics in breast cancer is rapidly evolving, and results from previous studies are encouraging. It can be expected that radiogenomics will play an important role in the future and has the potential to revolutionize the diagnosis, treatment, and prognosis of BC patients. This article aims to give an overview of breast radiogenomics, its current role, future applications, and challenges.

## Zusammenfassung

Die „Omics-Strategien“ – „genomics, transcriptomics, proteomics, metabolomics“ – haben viele potenzielle Anwendungsgebiete innerhalb der Onkologie und könnten das Verständnis der Krebsentstehung und des Fortschreitens der Erkrankung deutlich verbessern. Mit „Omics-Strategien“ können große Datenmengen verschiedenster biologischer Informationen schnell ausgewertet werden, mit dem Ziel, sinnvolle Biomarker für Brustkrebs und hiermit ein integratives Porträt dieser Erkrankung zu entwickeln. Diese neue Richtung in der medizinischen Wissenschaft wurde durch verschiedenste Fortschritte in Bildgebungs- und Bildanalysemethoden sowie die Entwicklung von Techniken zur Extraktion und Korrelation verschiedenster Bildgebungsparameter mit „Omics-Daten“ eingeläutet. Radiogenomics haben zum Ziel, Bildgebungscharakteristika (Phänotypen) mit Genexpressionsmustern, Genmutationen und weiteren genomassoziierten Eigenschaften zu korrelieren. Hiermit repräsentieren Radiogenomics die Evolution der Korrelation von Radiologie und Pathologie von der anatomisch-histologischen hin zur molekularen Ebene und stellen einen zentralen Schritt des „Omics-Paradigmas“ bei Brustkrebs dar. In Verbindung mit modernen Softwareanalysetechniken können quantitative und qualitative Bildgebungsbiomarker bisher beispiellose Erkenntnisse über komplexe Tumorbiologie liefern und ein besseres Verständnis der Krebsentstehung und -progression bewirken. Radiogenomics stellen ein sich rasch entwickelndes Forschungsfeld dar, und Resultate aus ersten Studien sind vielversprechend. Es ist zu erwarten, dass Radiogenomics zukünftig eine wichtige Rolle spielen werden, da sie das Potenzial haben, Diagnose, Behandlung und Prognose von Brustkrebs zu beeinflussen. Im vorliegenden Artikel wird ein Überblick über Radiogenomics der Brust, ihre aktuelle Rolle, zukünftige Anwendungen und Herausforderungen gegeben.

Within the field of oncology, “omics” strategies—genomics, transcriptomics, proteomics, metabolomics—have many potential applications and may greatly improve our understanding of the underlying processes of cancer development and progression. Omics strategies can play an important role in informing patient diagnosis, prognosis, and treatment [1, 2, 40]. In particular, they are naturally suited and highly promising for biomarker discovery as they allow for the rapid and simultaneous analysis of samples with rich biological information.

## Omics data in oncology

**Fig. 1:**
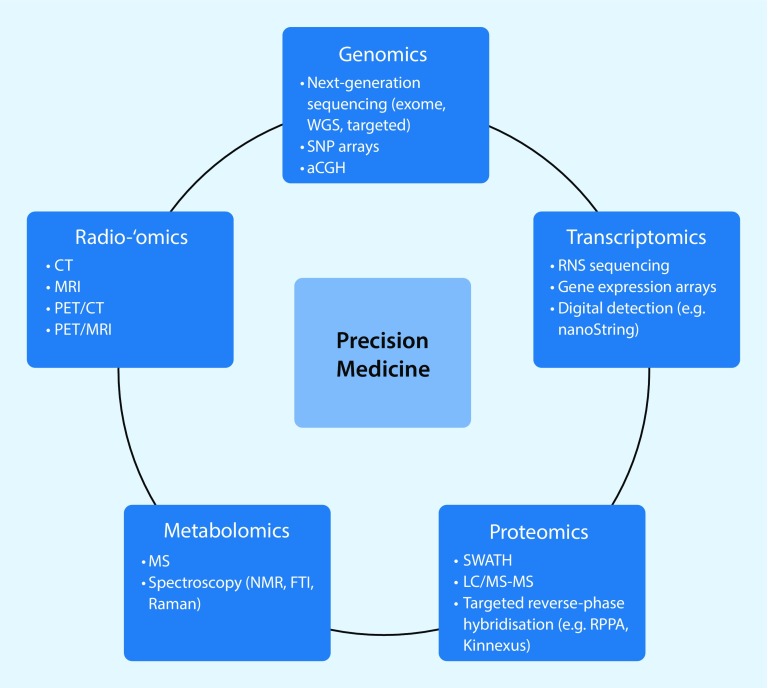
Omics technologies used in the characterization of breast cancer for precision medicine. Molecular profiling and imaging technologies currently used for ome examination are shown. *aCGH* array comparative genomic hybridization, *FTI* Fourier-transform infrared, *LC* liquid chromatography, *MS* mass spectrometry, *NMR* nuclear magnetic resonance, *RPPA* reverse phase protein array, *SNP* single nucleotide polymorphism, *SWATH* serial window acquisition of theoretical spectra, *WGS* whole-genome sequencing. (Modified with permission from [22], this content is not part of the Open Access licence)

In breast cancer (BC), the paradigm for omics strategies has always favored integrating multiple layers of omics data to achieve a complete portrait of BC. In the past decade, gene-expression profiling revolutionized BC classifications and replaced traditional categorizations based on immunohistochemistry with molecular subtypes (Fig. 1; [10, 11]).

Four intrinsic molecular subtypes of BC have been revealed from extensive profiling at the DNA, microRNA, and protein levels by The Cancer Genome Atlas (TCGA) Network [5]: luminal A, luminal B, HER2(human epidermal growth factor receptor 2)-enriched, and triple negative (TN). Molecular BC subtypes are unevenly distributed in patients, are associated with different tumor phenotypes, and have a distinct prognosis, response to treatment, preferential metastatic organs, and recurrence or disease-free survival outcomes [18]. Since 2011, the St. Gallen International Expert Consensus panel has used the molecular subtype-based recommendations for systemic therapies for BC [10, 11].

Patients with the luminal A subtype have the most favorable prognosis, followed by patients with luminal B, who have an intermediate prognosis. TN and HER2+ subtypes are associated with an unfavorable prognosis, but with the introduction of chemotherapy drugs such as trastuzumab and pertuzumab, the natural course of disease of TN and HER2+ has significantly improved [19]. Whereas luminal A cancers progress slowly over time with a greater chance of disease-free survival for patients [20], luminal B, HER2+, and TN BCs tend to recur, with a peak incidence of recurrence within the first 5 years for luminal B and the first 1–2 years for HER2+ and TN. Luminal cancers tend to metastasize to the bone while TN cancers metastasize to the viscera [33]. TN cancers are associated with a higher risk of regional relapse and the prognosis is dismal once the cancer spreads to regional lymph nodes, regardless of the number of nodes involved.

In the clinical and research settings, there is no readily available low-cost genetic testing to date and therefore molecular subtypes are commonly derived from invasive tissue sampling to guide therapy decisions. It should be noted that biopsies of small tumor regions are most likely not completely representative of the genetic, epigenetic, and/or phenotypic alterations of the entire tumor. In addition, although immunohistochemistry surrogates may provide clinical guidance, they have variable agreement with formal genetic testing (agreement rates have been reported to be between 41 and 100%) and are less robust for predicting patient outcomes [16]. Therefore, there is a strong argument for an alternative, more accurate means of differentiating molecular BC subtypes and elucidating the underlying processes of BC development and progression, which poses a tremendous and unique opportunity for advanced medical imaging.

In this review, we discuss the pivotal role of radiogenomics in BC within the larger omics paradigm in BC oncology. We aim to give an overview of breast radiogenomics, its current role, future applications, and challenges.

## Radiogenomics in breast cancer

Advances in medical imaging technologies, image analysis, and the development of high-throughput methods that can extract and correlate multiple imaging parameters with omics data have ushered in a new direction in medical research. Radiogenomics is a relatively new omics strategy that correlates imaging characteristics (i. e., the imaging phenotype) with underlying gene expression patterns, gene mutations, and other genome-related characteristics [4, 6, 9, 17, 26, 27, 32, 44].

Radiogenomics is not synonymous with radiomics, which is defined as the conversion of medical images to higher-dimensional, mineable data using computer classification algorithms and correlating these features with various data of interest such as patient characteristics, outcomes, and omics data for improved decision support [9, 25, 27, 32, 38].

Radiogenomics not only represents the evolution in the radiology–pathology correlation from the anatomical–histological level to the molecular level, but it is a pivotal step in the omics paradigm in BC for fully characterizing the disease. With the use of modern analytical software tools, discoveries of new quantitative and qualitative imaging biomarkers offer hitherto unprecedented insights into the complex tumor biology and facilitate a deeper understanding of cancer development and progression. In a typical radiogenomics study, multiple qualitative and/or quantitative imaging features—i. e., shape, size, volume, signal intensity, or texture—are manually or (semi-)automatically extracted from an imaging dataset and are then correlated with omics data. This correlation provides useful bidirectional information: Imaging parameters can be used to predict cancer genotypes, and imaging phenotypes can be predicted from gene signatures [4, 8, 26, 32].

In 2012 the first article on radiogenomics in BC was published followed by a growing body of literature ever since [49]. So far, the rapidly evolving field of radiogenomics in BC has almost exclusively focused on magnetic resonance imaging (MRI). MRI is an established tool in breast imaging, with multiple indications such as preoperative staging, monitoring of neoadjuvant chemotherapy, and screening of high-risk patients. Dynamic contrast-enhanced MRI (DCE-MRI) provides excellent morphologic information as well as limited functional information about abnormal vascularization as a tumor-specific feature. It is regarded as the most sensitive imaging modality for BC detection but has been criticized for its variable specificity. To add specificity and gain more functional information on BC, diffusion-weighted imaging (DWI) has been developed and found to be an essential addition to DCE-MRI in multiple studies [37]. Today, multiparametric (mp) MRI including DCE-MRI and DWI has been successfully implemented into clinical routine. Additional parameters such as chemical exchange saturation transfer (CEST), blood oxygen level-dependent (BOLD), hyperpolarized (HP) MRI, and lipid MP spectroscopy (MRSI) are currently being developed and investigated. These newer applications in MRI promise to provide additional functional information and may open up further avenues for radiogenomics research.

## Feature extraction approaches

For the purposes of radiogenomics analysis, imaging features can be extracted with human effort, semi-automatically or fully automatically using computer vision algorithms. Human feature extraction is based on image reading to provide specific variables such as lesion shape, margin, pattern, enhancement type, and kinetics; these features are defined by the American College of Radiology BI-RADS (Breast Imaging Reporting and Data System) MR lexicon. Human-extracted image variables are easily assessed but this process is time consuming and often limited by inter- and intra-observer variability, and thus semi- and fully automatic approaches should be preferred for feature extraction. While semi-automatic approaches still require human input in terms of tumor delineation or drawing the region of interest, fully-automatic computer-algorithm-extracted texture imaging features are of special interest for radiogenomics, quantifying the morphology and three-dimensional (3D) structure of the lesion of interest on a voxel-by-voxel basis.

Texture features are evaluated by texture analysis, which comprises four tasks with the aim of quantifying the morphology and internal structure of the tissue: feature extraction, texture discrimination, texture classification, and shape reconstruction [42]. In feature extraction, a numerical value is calculated based on statistical, structural, or model-based processing, e. g., with publicly available software such as the open source software MaZda (Technical University of Łodz, Institute of Electronics, Łodz, Poland; http://www.eletel.p.lodz.pl/programy/mazda/). In texture discrimination, images are segmented and regions with similar texture features are grouped together. These regions can be matched on predefined characteristics such as amount of fibroglandular tissue, benign tissue, or malignant breast lesions. The derived information can then be used to reconstruct 3D shapes and models and finally be correlated with genomic signatures or outcome variables.

In addition, data-mining algorithms can be used to extract dynamic variables such as enhancement kinetics, which allows for the assessment of neoangiogenesis as a tumor-specific feature. Kinetic features that are usually evaluated are the rate of enhancement on early postcontrast-enhanced sequences, peak enhancement, and late postcontrast-enhanced sequences.

Although computer vision algorithms are of special interest because they can facilitate the assessment of large data volumes, are not reader-dependent, and can provide information that is beyond human perception, they are not ready to be introduced into clinical routine, as research data are not yet fully reproducible owing to a lack of image protocol and data standardization.

## Radiogenomic approaches

In exploratory radiogenomics studies, the extracted imaging features are tested against multiple different genomic characteristics, while metrics such as the false discovery rate are often used to detect meaningful prospective variables [12, 36, 39]. Hierarchical clustering is a method for evaluating similarities in large datasets and has been used famously in the original definition of the molecular subtypes of BC by Perou et al. [35]. In this approach, individual data points that show similarities are clustered until the relationship between all data points is established. The largest group at the top of the map is then used to define all groups within the dataset.

In hypothesis-driven radiogenomics studies, imaging characteristics are correlated with specific genetic signatures [26] with many potential benefits for BC diagnosis and therapy. As mentioned earlier, no low-cost genetic testing is available to date and the development of surrogates by means of radiogenomics with medical imaging is of great interest. In addition, radiogenomics might be used to develop imaging biomarkers to predict outcome parameters, such as therapy response or metastases [3].

## Current applications

Thus far, MRI radiogenomics in the breast has mainly focused on DCE-MRI and the analyses of individual genomic signatures, BC molecular subtypes, or clinically used recurrence scores, with promising results.

### Individual genomic signatures

In 2012, Yamamoto et al. conducted the first radiogenomics study in BC, demonstrating in ten BC patients that radiogenomics can be used to correlate gene expression patterns with imaging features in DCE-MRI [49]. In this groundbreaking study, the authors showed that 21 of 26 imaging characteristics were significantly associated with 71% of approximately 52,000 variably expressed genes. They found that 12 imaging characteristics were significantly correlated with BC genes, while 11 were significantly correlated with prognostic molecular characteristics. In a follow-up study by the same investigators using computer vision-extracted features and RNA sequencing, the enhancing rim fraction score was significantly associated with early metastasis [48].

Another group of authors, Zhu et al., investigated potential correlations of DCE-MRI features such as tumor size, shape, and morphology with genomic features such as transcriptional activities, protein expressions, and mutations for 91 breast carcinomas [51]. All selected DCE-MRI characteristics were associated with transcriptional activities of pathways, in particular tumor size, indicating that upregulated pathways are more common in large cancers. At the same time, associations between transcriptional activities and blurred tumor margins and irregular shape were found, indicating more aggressive malignancies.

### Molecular breast cancer subtypes

**Fig. 2:**
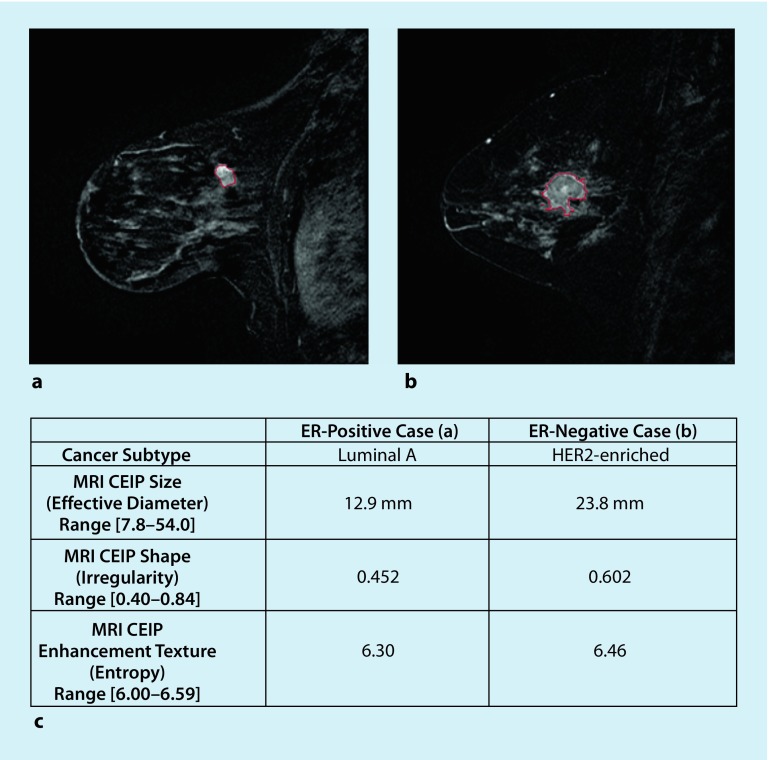
Example cases including segmentation outlines obtained from the computer segmentation method. **a** ER-positive example; **b** ER-negative example; **c** CEIP values (and ranges) for size, irregularity, and enhancement texture of two example cases. *CEIP* computer-extracted image phenotypes, *ER* estrogen receptor, *HER2* human epidermal growth factor receptor 2, *MRI* magnetic resonance imaging. (Reprinted with no modifications under a creative common license (https://creativecommons.org/licenses/by/4.0/) from [29])

As shown in recent efforts, radiogenomics has the potential to identify imaging biomarkers as reliable surrogates for genetic testing in the future. Grimm et al. found strong correlations between morphologic, kinetic, and textural imaging findings and luminal A and B subtypes [15]. While the classifier model by Waugh et al. had limited success, achieving an accuracy of 57.2% [46], Li et al. evaluated a classifier model utilizing tumor phenotypes to distinguish between molecular subtypes with promising results (Fig. 2; [29]).

While the following are not radiogenomics studies, they show that imaging features are associated with molecular subtypes. In a recent study involving 278 cancer patients, Grimm et al. found significant correlations between DCE-MRI BI-RADS descriptors and molecular subtypes [14]. Previous studies have also reported associations between DCE-MRI enhancement kinetics and molecular BC subtypes. For instance, Elias et al. demonstrated that the luminal B subtype is associated with a higher internal enhancement of the tumor, while HER2-enriched cancers are more likely to show fast initial enhancement or wash-out kinetics [7]. HER2 subtypes have been described as being associated with a circumscribed margin, while TN subtypes are associated with rim enhancement and high T2 signal intensity [13, 45]. In DWI, the highest apparent diffusion coefficient (ADC) values were found in HER2-enriched tumors, while luminal B/HER2-negative cancers showed the lowest ADC values [23, 31, 34], which might be due to the increased vascularization found in HER2-positive subtypes. These findings indicate that the assessment of functional tumor parameters with radiogenomics can be expected to contribute to our deeper understanding of BC biology.

### Recurrence scores

**Fig. 3:**
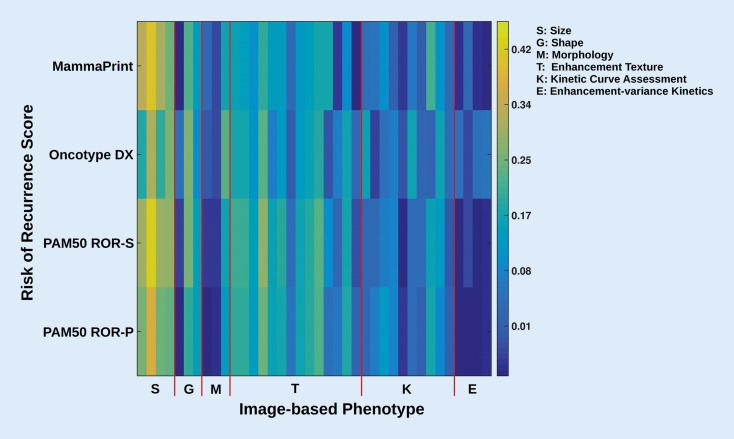
Color map showing the correlation of MRI-based phenotypes with the recurrence predictor models of MammaPrint, OncotypeDX, PAM50 ROR-S, and PAM50 ROR-P. Here, *yellow* indicates higher correlation than *blue*. The different gene assays (recurrence predictor models) serve as “reference standard” in this study. *ROR-P* risk of relapse based on proliferation, *ROR-S* risk of relapse based on subtype. (Reprinted with permission from [28], this content is not part of the Open Access licence)

**Fig. 4:**
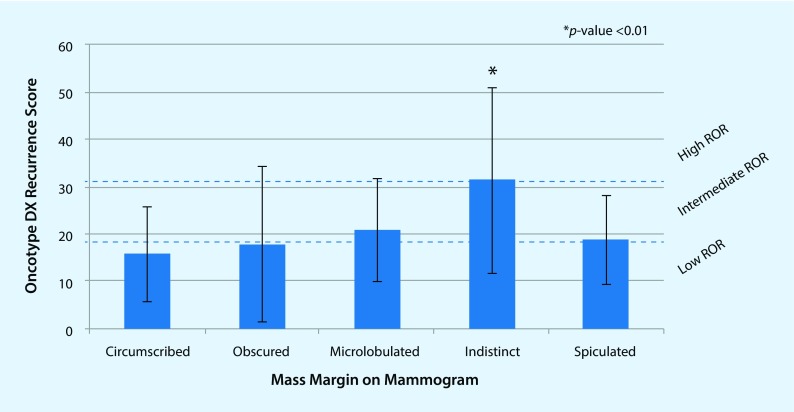
Bar chart shows average ODxRS by using mammographic mass margins. *Asterisk* Indistinct mass margins versus all other mass margins; *p* value adjusted for age at detection and personal history of invasive carcinoma or DCIS. ROR: low (score <18), intermediate (score 18–30), and high (score ≤31). *DCIS* ductal carcinoma in situ, *ODxRS* Oncotype DX test recurrence score, *ROR* risk of recurrence. (Reprinted with permission from [47], this content is not part of the Open Access licence)

Another clinically relevant application of radiogenomics is the correlation of imaging characteristics with prognostic genomic assays that provide scores for the risk of recurrence and are used to guide treatment decisions. Ashraf et al. demonstrated that DCE-MRI features indicative of greater tumor vascularization were associated with an increased risk of cancer recurrence [3]. Sutton et al. developed a model incorporating imaging and pathological information that showed a correlation with the OncotypeDx recurrence score [41]. In another study, Li et al. evaluated whether computer-extracted imaging phenotypes could predict cancer recurrence using multigene assays, indicating that larger, more heterogeneous tumors have a higher risk for recurrence [28]. In this study, significant associations between BC MRI radiomics features and recurrence scores, especially MammaPrint, OncotypeDx, and PAM50/Prosigna, were found (Fig. 3). In a very recent study, Woodard et al. evaluated the association of BI-RADS mammography and MRI features with BC recurrence in estrogen receptor (ER) positive patients using the OncotypeDx assay [47]. They found indistinct mass margins and fine linear branching calcifications to be significantly associated with a higher recurrence score, while breast density was inversely associated with the recurrence score (Fig. 4). These studies illustrate that radiogenomics has the potential to identify multiple imaging biomarkers of BC recurrence risk, with larger studies needed to validate these preliminary findings.

## Challenges and future perspectives

One of the main challenges of radiogenomics is the generation of big data, which must be stored, managed, and analyzed in a standardized, cost-effective way. Initiatives such as the Center for Advancing Translation Science of the National Institutes of Health (NIH, 2011) are being developed to address this problem. In addition, research in the field of radiogenomics is still limited by the inter- and intra-institutional data heterogeneity caused by different hardware, scan protocols, and postprocessing. Furthermore, genetic testing is challenging and costly, while the availability of genetic data is generally limited. The TCGA and Cancer Imaging Archive have been launched to store imaging and genetic data derived from different institutions. However, owing to these challenges, the conclusions that can be drawn from radiogenomic BC studies are limited by their mostly retrospective nature and small patient cohorts. To date, the evolving field of radiogenomics in breast imaging has almost exclusively focused on DCE-MRI, while most studies aimed to correlate genomic features with cancer subtypes and recurrence scores.

However, the field of imaging biomarkers development with MRI is rapidly growing. In DWI advanced techniques such as intravoxel incoherent motion, stretched exponential DWI, and DW kurtosis imaging are being investigated and hold promise for providing additional robust imaging biomarkers that can be incorporated in radiogenomic studies [30]. In addition, other MRI techniques that may be used for radiogenomic research include spectroscopy (proton, phosphorus, lipid), sodium imaging [50], CEST imaging [24], BOLD [21], and arterial spin labeling MRI [43]. Radiogenomics research in BC is still in its infancy. Larger prospective studies utilizing the full wealth of information that MRI can offer and considerable efforts in standardization and quality control are warranted, especially regarding outcome-related data, to meaningfully implement radiogenomics in the clinical setting.

## Practical conclusion

- Radiogenomics examines the correlations of imaging phenotypes with characteristics derived from omics strategies—genomics, transcriptomics, proteomics, metabolomics.
- The integration of radiogenomics is a pivotal step toward completing the omics paradigm in BC.
- Thanks to the noninvasive nature and ubiquitous use of medical imaging in clinical routine, radiogenomics can elucidate disease processes by adding to our understanding of the disease etiology and helping to determine patient diagnosis, prognosis, and treatment.
- Exploration of additional functional imaging data in conjunction with omics technologies will open new avenues of multidimensional radiogenomic research.
- The implementation of radiogenomics in clinical BC care can further enhance the role of radiology. Additional efforts, rigorous standardization, and quality control are needed to validate already described radiogenomic correlations, discover new correlations, and define clinically relevant imaging biomarkers.
